# Epigenetic factors in breast cancer therapy

**DOI:** 10.3389/fgene.2022.886487

**Published:** 2022-09-23

**Authors:** Runjhun Mathur, Niraj Kumar Jha, Gaurav Saini, Saurabh Kumar Jha, Sheo Prasad Shukla, Zita Filipejová, Kavindra Kumar Kesari, Danish Iqbal, Parma Nand, Vijay Jagdish Upadhye, Abhimanyu Kumar Jha, Shubhadeep Roychoudhury, Petr Slama

**Affiliations:** ^1^ Department of Biotechnology, School of Engineering and Technology, Sharda University, Greater Noida, India; ^2^ Dr. A.P.J Abdul Kalam Technical University, Lucknow, India; ^3^ Department of Biotechnology, School of Applied and Life Sciences (SALS), Uttaranchal University, Dehradun, India; ^4^ Department of Biotechnology Engineering and Food Technology, Chandigarh University, Mohali, India; ^5^ Department of Civil Engineering, Netaji Subhas University of Technology, Delhi, India; ^6^ Department of Civil Engineering, Rajkiya Engineering College, Banda, India; ^7^ Small Animal Clinic, University of Veterinary Sciences Brno, Brno, Czechia; ^8^ Department of Applied Physics, School of Science, Aalto University, Espoo, Finland; ^9^ Department of Medical Laboratory Sciences, College of Applied Medical Sciences, Majmaah University, Al Majma'ah, Saudi Arabia; ^10^ Health and Basic Sciences Research Center, Majmaah University, Al Majma'ah, Saudi Arabia; ^11^ Center of Research for Development (CR4D), Parul Institute of Applied Sciences (PIAS), Parul University, Vadodara, Gujarat; ^12^ Department of Life Science and Bioinformatics, Assam University, Silchar, India; ^13^ Department of Animal Morphology, Physiology, and Genetics, Faculty of AgriSciences, Mendel University in Brno, Brno, Czechia

**Keywords:** cancer, breast, epigenetics, estrogen, therapy

## Abstract

Epigenetic modifications are inherited differences in cellular phenotypes, such as cell gene expression alterations, that occur during somatic cell divisions (also, in rare circumstances, in germ line transmission), but no alterations to the DNA sequence are involved. Histone alterations, polycomb/trithorax associated proteins, short non-coding or short RNAs, long non—coding RNAs (lncRNAs), & DNA methylation are just a few biological processes involved in epigenetic events. These various modifications are intricately linked. The transcriptional potential of genes is closely conditioned by epigenetic control, which is crucial in normal growth and development. Epigenetic mechanisms transmit genomic adaptation to an environment, resulting in a specific phenotype. The purpose of this systematic review is to glance at the roles of Estrogen signalling, polycomb/trithorax associated proteins, DNA methylation in breast cancer progression, as well as epigenetic mechanisms in breast cancer therapy, with an emphasis on functionality, regulatory factors, therapeutic value, and future challenges.

## Introduction

The greatest serious hazard to women’s health in developed countries is breast cancer. Breast cancer is the most frequent cancer in women around the world (Bray et al., 2004; Chen et al., 2016) and distant metastasis is the major cause of poor survival (Gupta and Massague, 2006; Hanahan and Weinberg, 2011). The most common cause of death among breast cancer patients is metastasis. The molecular pathways that drive tumour cells to become metastatic have been thoroughly investigated, leading to major advances in prediction and treatment techniques. However, the significant high percentage of breast cancer related fatalities continues to be a major source of concern. As a result, elucidating novel metastasis-related molecular processes is critical for improving breast cancer therapy outcomes.

Since 2004, invasive breast cancer has been on the rise, in 2018, more than two million instances were reported around the world and more than 270,000 instances projected in the United States by 2020.

It is vital to completely understand the molecular pathways that enable breast cancer cell metastasis in order to create strategies to improve breast cancer patient survival and prognosis. Long non-coding RNAs (lncRNAs) have subsequently been revealed to play an important role promoting breast cancer metastasis through a variety of molecular pathways, albeit their exact functional characteristics have yet to be defined. Long noncoding RNAs (lncRNAs) have recently been linked to breast cancer metastasis in a number of studies (Bin et al., 2018; Klinge, 2018; Zhou et al., 2018; Arun and Spector, 2019; Tomar et al., 2020). Long noncoding RNAs are noncoding RNAs with a size of more than 200 nucleotides that have a role in a range of biological processes, particularly cancer cell invasion.

In transformed cells, epigenetic modifications include changes in DNA methylation, such as global hypomethylation or altered histone tail modification patterns, locus specific hypermethylation, as well as nucleosomal remodelling. DNA methylation is defined by Hinshelwood and Clark (Hinshelwood and Clark, 2008) (2008) as an enzyme-driven chemical modification to DNA sequence that happens most frequently at CpG dinucleotides among mammals.

DNA hypomethylation has been linked to gene reactivation and chromosomal instability, which can result in proto-oncogene overexpression, Imprinting loss, skewed or missing X-chromosomal inactivation, and increased recombination and mutation rates (De Smet et al., 2004). Gene suppression and genomic instability are connected to DNA hypermethylation as well as the suppression of tumour suppressor genes. In humans, PCDHB15 is a member of the cadherin superfamily of calcium-dependent cell-cell adhesion molecules that encodes for the PCDHB15 protein. CDH1 (also known as E-cadherin) and other cell adhesion molecules operate as epithelial-mesenchymal transition suppressors. In this CDH1 epigenetic silencing has been reported often in human cancer cases, including breast cancer (de Ruijter et al., 2020).

Another epigenetic process that can regulate gene expression by altering chromatin shape is post-translational histone tail modifications, which are linked to DNA methylation (Martin and Zhang, 2005; Baylin and Ohm, 2006). Furthermore, it has been demonstrated that several nucleosomal remodelling regulators are also engaged in DNA methylation and histone modification regulation (Esteller et al., 2000; Bird, 2002; Martin and Zhang, 2005; Baylin and Ohm, 2006). Understanding all of these epigenetic modifications and their role in breast carcinogenesis is critical for further advancements in breast cancer detection, prognosis, and treatment.

In the presence of the dinucleotide sequence CpG, DNA hypermethylation is a post-replication alteration that nearly exclusively affects cytosines’ pyrimidine ring (Pfeifer and Besaratinia, 2009). In mammalian genomes, the bulk of CpG dinucleotides (75%) are methylated. The quantity of 5-methylcytosine in 1% of all bases varied somewhat between tissue types. Repeating elements and transposons, which constitute roughly one-third of the human genome, include more than 90% of all methylation cytosines. Owing to the inherent carcinogenic potential of methylated cytosine residues, the proportion of Nucleotide bases in the genomes has been lowered over time, resulting in reduced number of CpGs than the quantitatively expected.

Cytosines that are methylated are more vulnerable to endogenous or exogenous mutagenesis mechanisms than other DNA bases, with CpG site mutation rates projected to be higher than other transitional mutations (Jones et al., 2008). Transitions from C to T at CpG dinucleotides account for almost a 1/3 of all known germ line and somatic and mutations, albeit the distribution varies depending on the tumour type (Lo and Sukumar, 2008). CpG islands are tiny DNA fragments (ranging in size from 200 base pairs to several kilo base pairs) found in 60% of all genes.

**FIGURE 1:**
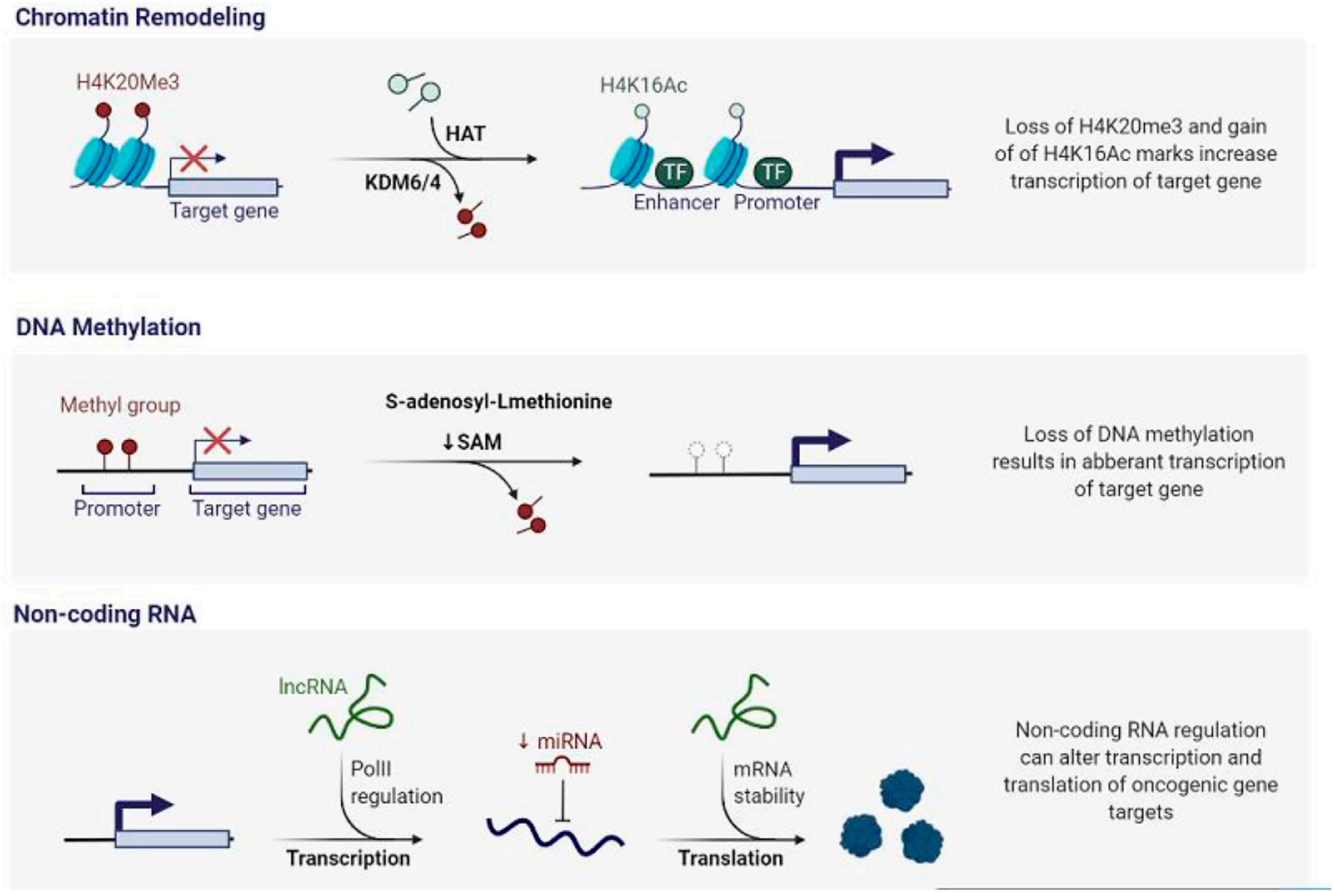
Epigenetic Deregulation in Cancer. A vast number of epigenetic modifiers are mutated or activated inappropriately during cancer genesis. Simultaneously, epigenetic alterations such as DNA methylation, histone modifications, and microRNAs cause aberrant gene expression, resulting in genomic instability.

CpG islands that are ordinarily unmethylated in cancer cells may become methylated, potentially silencing critical genes such as tumour suppressor genes. At the same time, due to insufficient transcriptional regulation of typically silent genes like oncogenes or retrotransposons, CpG dinucleotides in other places can become unmethylated. DNA methylation silences tumour suppressor genes (TSGs) that govern tumor development, DNA repair genes, oestrogen receptor genes, or genes that regulate angiogenesis. Because transcription factors which interface with methylated DNA differ from those that interact with unmethylated DNA, DNA methylation has an impact on gene expression (Figure 1) Hypermethylation of promoter regions silences the gene, which is a critical step in carcinogenesis with substantial implications for cancer prevention.

In human malignancies and primary tumours, some tumour suppressor genes and other malignant genes have been discovered to be hypermethylated (Frigola et al., 2006). Cell -cycle control, DNA repair, cell death, cellular maintenance, and invasion are among their physiologic functions. Table 1 shows the genes which are most commonly methylation in breast cancer. Epigenetic cancer research has taken on a new dimension with the finding of long-range gene silence induced by epigenetic alterations (Stransky et al., 2006). Long-range epigenetic silencing appears to be ubiquitous during carcinogenesis, according to a recent study that revealed transcriptional dysregulation that can be regulated by epigenetic processes (Stransky et al., 2006).

**TABLE 1:** Breast cancer genes that are hypermethylated.

Genes	Function
BRCA1 Wu et al. (2010)	DNA damage repair
APC Saelee and Pongtheerat, (2020)	Catenin, cell proliferation, migration, and adhesion inhibitor
GSTP1 Kanwal et al. (2014)	Prevention of oxidative DNA damage by conjugation to glutathione
Cyclin D2 Evron et al. (2001)	Regulators of CDK kinases
PTEN Ramadan and Hashmin, (2021)	Regulating the AKT/PBK signalling pathway negatively
*p*16^*INK*4*α*^ Hui et al. (2000)	Cyclin-dependent kinase inhibitor
*RASSF1A* Li et al. (2019)	Ras effector homologue, cell cycle arrest
RARβ Wang et al. (2020)	Retinoic acid receptor
ZMYND10 Wang et al. (2019)	Inhibitor of cancer cell colony formation

### Epigenetically regulated genes in breast cancer

Several research have sought to investigate the role of hypermethylation of TSG genes’ promoters in breast cancer, as well as the relationship between methylation of certain CGIs in TSGs and a variety of breast cancer clinical states. Table 1 lists the most important hypermethylated genes implicated in breast cancer functions so far. Methylation of these TSG promoters is linked to cancer cells losing all TSG protein products and developing a malignant phenotype. This DNA hypermethylation is a reversible signal, possibly due to the activity of Demethylase, which reverses the reaction of DNA methyltransferase and is a strong contender to be one of its key partners in shaping genome methylation patterns (Strahl and Allis, 2000; Ito et al., 2002). As a result, many recent research has focused on a novel strategy to cancer treatment that aims to block DNA hypermethylation and/or re-expression of silenced TSGs.

To create the transcriptional regulatory platform, DNA is packed into chromatin, a highly structured and dynamic protein–DNA complex. Histone modifications and composition interact with the binding of a variety of nonhistone proteins to control open (euchromatin) and closed (heterochromatin) chromatin states. The nucleosome is chromatin’s most basic component, wrapping 146 bp of DNA around an octamer made up of four core histones, an H3/H4 tetramer, and two H2A/H2B dimers (Strahl and Allis, 2000; Ito et al., 2002). The importance of local chromatin architecture in the regulation of gene expression is now widely acknowledged. Posttranslational changes to the N terminal tails of histones have a big impact on chromatin architecture.

Methylation, acetylation, phosphorylation, ubiquitination, sumoylation, ADP ribosylation, deamination, and proline isomerization are all covalent alterations to core histones (Schubeler et al., 2004; Shilatifard, 2006). The discovery of multiple histone modifications with varied functions in gene regulation aided the identification of a regulatory histone code, that defines at least partially overall transcriptional possibilities of a gene or genomic region (Xu et al., 2009). Altering the N terminus histone tail, which affects nucleosome density and positioning, enables this packed, inaccessible DNA accessible to DNA binding proteins during gene transcription initiation (Ito et al., 2002). Each histone modification acts as a chromatin organisation signal. Histone acetylation (hyperacetylation) is associated with increased transcriptional activity, whereas hypoacetylation (hypoacetylation) is associated with gene repression (Forsberg and Bresnick, 2001; Wade, 2001). Transcription related Protein (, p53, p73, E2F1, STAT1, GATA1, HMGB1, YY1, and NFkB etc), hormone response (GR, ER, and AR), nuclear transporter (Importin7), WNT signalling (catenin), DNA repair (Ku70) and heat shock/chaperone reaction (HSP90) are examples of HDAC substrates (Bolden et al., 2006; Kim et al., 2006).

For a long time, it was considered that methylation cytosines on DNA and deacetylated histones were two separate processes that could regulate chromatin structure and gene expression independently (Turner, 2000; Roth et al., 2001). HDACs have been linked to Epigenetic modifications by methyl group associated protein like MeCP2, which may read methylated sites on DNA and attract HDACs to them, or by HDACs directly interacting with DNA methyltransferases (Lo and Sukumar, 2008) (DNMTs). (Robertson et al., 2000; Bachman et al., 2001). Histone H3 lysine nine gets acetylated in functional chromatin regions, but when a genes is silenced, it becomes methylated, creating a binding domain for hetero-chromatin protein1 (HP1) (Litt et al., 2001; Peters et al., 2002). When serine 10 is phosphorylated, another epigenetic change, phosphorylation, prevents lysine nine from becoming methylated (Rea et al., 2000). PolyADP ribosylation is another alteration. PolyADP ribosylation has really been reported to affect chromatin structure through two methods, either covalently, by establishing short chains of Adenosine Diphosphate ribose polymers to histone proteins, or non-covalently, thereby attracting histones to the extended and branching polymers.

### Epigenetic mechanism in breast cancer therapy

Diagnostic and prognostic methods based on epigenetics play an important role in precision medicine. Precision oncology benefits substantially from epigenetics-based diagnostic and prognostic techniques. Numerous DNA methylation diagnostic tests, in particular, are now being tested in clinics or are already in use. Precision oncology efforts to address dysregulated epigenetic pathways resulted in the development of epidrugs, or drugs that target epigenetic modulators. The FDA has approved only nine epidrugs, many more are in clinical studies for solid and hematological tumours (NCT01928576, NCT03179943), including antagonists of DNA methyltransferases (DNMTs) (NCT03164057, NCT02717884), EZH2, IDH and HDAC.The phase II trials (NCT00676663 and NCT00828854) exploring the epidrugs’ effectiveness when used in conjunction with normal treatment in oestrogen receptor positive (ER+) breast cancer are significant, reflecting recent developments in the knowledge of the epigenetic process. Due to ER expression, over 80% of all affected individuals are classified as ER+, and over 90 percent of all these patients have a 5 year cumulative rate of survival. Endocrine-based therapies such as ER-blockade, oestrogen synthesis inhibition, and selective ER degradation are used to treat most ER + cancers since ER is the primary oncogenic driver (Zardo et al., 2003; Thomas and Potter, 2013).

### Long non coding RNAs

LncRNAs are RNA molecules having a length of more than 200 nucleotides but no apparent protein-coding function. Over 10,000 lncRNAs have been identified in the human transcriptome, with their genes located inter- or intra-genes in the genome. However, only a few have been thoroughly described. RNA polymerase II transcribes LncRNA genes, which then go through 5′ capping, splicing, 3′ cleavage, and polyadenylation. LncRNA loci are comparable to those of protein-coding genes at the chromatin level, although they frequently lack introns or have one or two. Splicing matures lncRNAs in the same way that it matures pre-mRNAs. n general, lncRNAs are found in the nucleus, although they have also been found in the cytoplasm and exosomes, and their expression levels are often lower than those of coding genes. Many investigations have found that their expression differs depending on the cell type24. In compared to protein-coding genes, lncRNAs are under low selected pressure, but their selective pressure is stronger than genomic repeat sequences. When comparing the sequences of lnRNAs from different species, brief highly conserved sequences can be found, demonstrating that they have preserved information about their cellular location and structure during evolution (Shang et al., 2000; Métivier et al., 2003; Moore and Proudfoot, 2009; Derrien et al., 2012; Rinn and Chang, 2012).

Following is a description of the lncRNAs H19, TINCR, MALAT, and NEAT1 DANCR, whose aberrant expression is linked to the growth and metastasis of BC.

### H19 LncRNA

It has been established that BC development and dysregulated long non-coding RNA H19 (H19) expression are related (Yang et al., 2016; Hu et al., 2018). Over 70% of BC tumours, including ER+ and ER-, HER2+ and HER2-positive tumours, have highly expressed H19 (Yang et al., 2016; Wang et al., 2018). This lncRNA has greater expression in BC for a number of usual mutational polymorphisms as well (Vennin et al., 2017; Wang et al., 2018). Apoptosis suppression and cell proliferation promotion are two biological reactions in which the Akt signalling pathway is involved (Yang et al., 2016). The maternal allele encodes H19, a 2.3-kb lncRNA that is regarded as an oncogene in several malignancies. At the H19/IGF2 locus, a novel lncRNA called 91H is being produced in the H19 antisense direction. In breast cancer, the 91H lncRNA is in charge of preserving the genomic imprinting of the H19/IGF2 locus by preventing histone and DNA methylation on the maternal allele (Hu et al., 2018; Wang et al., 2018). E2F1 stimulates H19, which aids the G1-S transition in breast cancer cells (Vennin et al., 2017). Through the activation of Akt, the miR-675 produced by H19 downregulates the c-Cb1 and Cb1-b proteins and activates EGFR and c-Met to encourage cell growth (Berteaux et al., 2005; Zhang et al., 2017) discovered that overexpression of the lncRNA MEG3 inhibits cancer growth in a mouse model of breast cancer by inhibiting Akt signalling, in addition to causing cell cycle arrest in the G0/G1 phase. (Chen et al., 2017). demonstrated that lncRNA PTENP1 restricts the growth of breast cancer cells by downregulating the MAPK and AKT signalling pathways.

### TINCR lncRNA

In 2018 (Liu et al., 2018), it was found that TINCR lncRNA (TINCR) influences how primary BC tumours develop and how they spread later. In a certain study of 24 patients, the qPCR technique identified greater TINCR BC expression compared to non-BC participants. Additionally, SP1-zinc finger transcriptional factor, which normally identifies the Guanine Cytosine -rich sequences in promoter regions, causes greater TINCR activity (Liu et al., 2018).

### MALAT lncRNA

Multiple BC types have abnormal expression of the lncRNA MALAT (MALAT), and this abnormal expression is associated with metastasis and a poor prognosis (Jadaliha et al., 2016; Wu et al., 2019). Further evidence suggests that high concentrations of 17- oestradiol can impede this lncRNA activity (Zhao et al., 2014). In a fascinating study, individuals with early post-BC-resection fever had higher MALAT levels (Li et al., 2018), which was associated with a worse prognosis. Additionally, MALAT deletion in mouse 4T1 xenografts markedly reduced inflammation and the lung metastases that are frequently observed in BC (Li et al., 2018).

### NEAT1 lncRNA

NEAT1 is a crucial oncogene in cancer and has a big impact on BC’s ability to induce EMT (Lu et al., 2016). In a sample of 179 BC patients, abnormal NEAT1 activity influenced chemoresistance and cancer cell stemness, and it has been expressed 6.86 times greater in BC patients than that in 192 controls (Shin et al., 2019).

### Estrogen subtypes and ER signalling pathways

Estrogen promotes a variety of developmental processes in the body, involving reproductive maturity and bone growth, as well as energy balance via glycaemic control, intake rate, and thermoregulation. Estrogen also regulates mammary gland development through coordinating mitogenic and epigenetic processes. Several chemicals, as well as naturally occurring substances like polyphenols, which serve as a hypermethylation agent, can reverse the epigenetic silencing of tumour suppressor genes (Mathur and Jha, 2020). .Estrone, 17-Estradiol,Estriol, Estetrol (i.e, E1,E2,E3,E4) & Estrone-sulfate are the five major oestrogen subtypes (E1s). E1 and E2, the body’s two major estrogens, are reversibly transformed to E2, the physiologically active variety. Only E3 and E4 are identified throughout pregnancy, with E3 being the most prevalent. Because steroid sulfatases convert it to its active metabolite, E1 and E2, *in situ*, E1s is largely employed as an oestrogen reservoir (Mukherjee et al., 2017).

### Epigenetic mechanism underlying Erα signalling

Epigenetic mechanisms are involved in ER signalling. In response to E2 stimulation, multitudes of ER co-regulators are transported to chromosomes in a synchronised way to ensure appropriate transcriptional and repressive activity at ER target sites. Regardless of the fact that every ER molecule usually stays on the chromatin for few moments at most, ER has been observed cycling on and off the chromatin for minutes and hours after E2 stimulation (Djebali et al., 2012; Johnson and O’Malley, 2012; Paakinaho et al., 2017; Wils and Bijlsma, 2018; Zhang et al., 2020). PRMTs, the SWI/SNF complex, P300/CBP & the Mediator complex, as well as the p160 family of proteins, are all significant epigenetic ER coactivators. Members of the p160 family the co—activators i.e., SRC-1, SRC-2, and SRC-3, bind to ER directly and act as a recruiting platform for other activating enzymes and proteins to be recruited by ER to change chromatin, including chromatin remodelling complexes Breast cancer messes up epigenetic mechanisms that are essential for mammary gland development. The mammary gland’s balance self-renewal, and tissue integrity is regulated by a variety of signalling cascades and chromatin moderators, and also hormonal factors. Embryonic, pubertal, & reproductive stages are all three stages in the development of the mammary gland. WNT and Hedgehog (HH) signalling pathways coordinate embryonic mammary gland development, whereas hormones control pubertal and reproductive stages (Suzuki et al., 2008).

The reactivation of various developmental pathways, which would be a common characteristic of many malignancies, is connected to the longevity of a mammary gland stem population of cells in cancer patients (Xiang et al., 2013). Derailment of important epigenetic mechanisms during breast development currently plays an essential role in the pathogenesis of breast cancer, according to research conducted in the last few years with the introduction of technical breakthroughs like as next-generation sequencing. This article discusses how the functional relationship between epigenetic alterations and developmental signalling cascades contributes to breast cancer.

### WNT signalling epigenetic modification in ER + breast cancer

WNT signalling abnormalities have been associated to the onset and progression of a variety of cancers, including breast cancer. Breast cancer is aided by epigenetic suppression of WNT antagonist genes such as SFRP and DKK37. Chronic WNT signalling in breast cancer, which is linked to a poor prognosis, is caused by the methylation of these genes, which silences them (Bell et al., 2019). As a result of these changes, catenin stays constitutively active, resulting in enhanced stem cell replacement and division, which has been linked to disease resurgence (Serrano-Gomez et al., 2016). One of the constituent of the DKK family, i.e DKK3 had significantly more promoter methylation in tumours from individuals with lymph node metastases, advanced stage disease, or breast cancer samples with positive ER status. status of mammary cancer samples.

**FIGURE 2:**
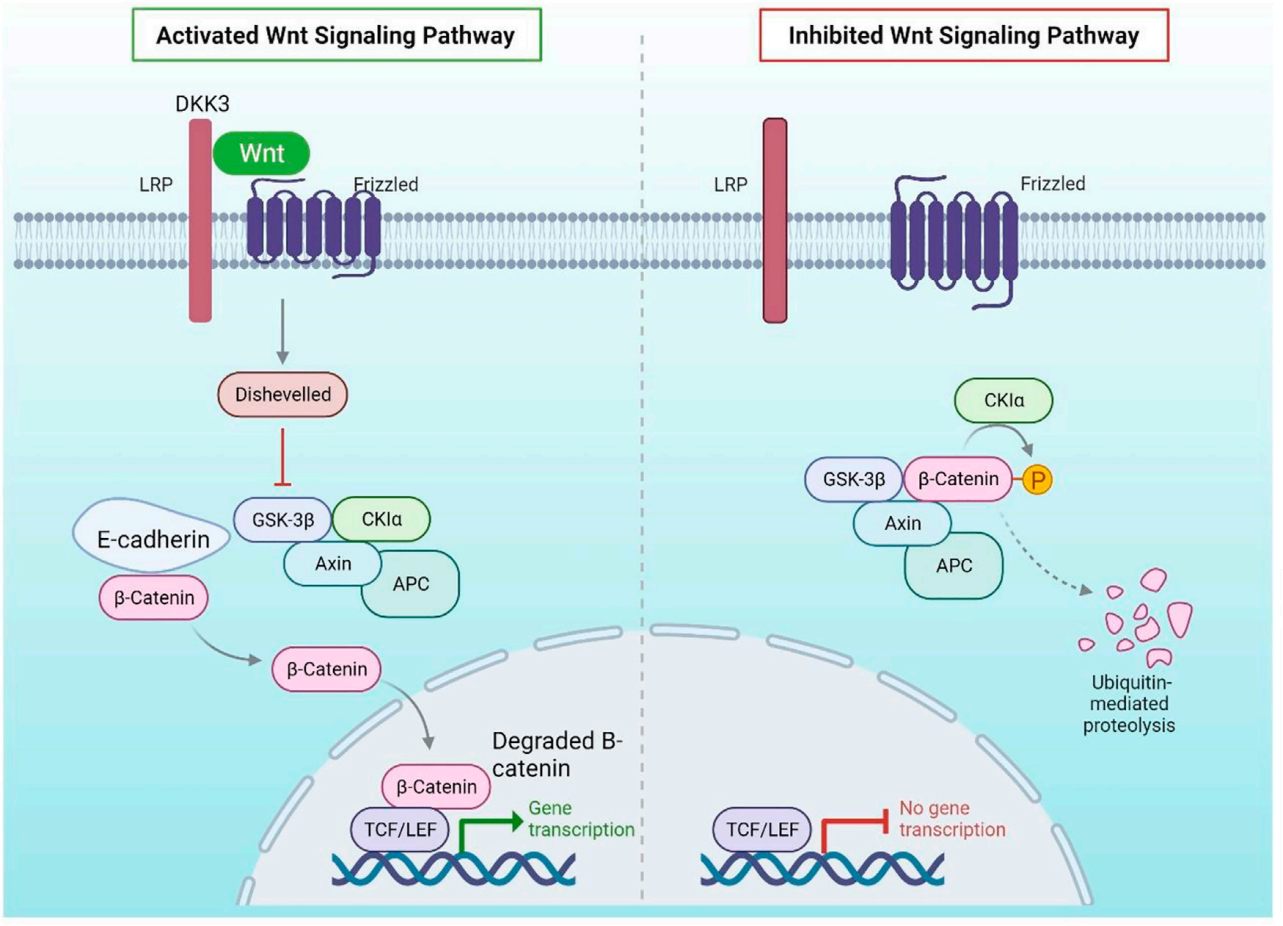
Wnt Signalling Pathway: DKK3 binds to LRP, a WNT pathway coactivator of Frizzled, in normal mammary epithelial cells, preventing the pathway from being activated in the presence of the WNT ligand. In the absence of WNT activation, E-Cadherin binds to cytoplasmic -catenin, which is destroyed by GSK3. The DKK3 promoter, on the other hand, is hypermethylated in breast cancer, resulting in its downregulation. LRP can coactivate Frizzled in the presence of the WNT ligand in the absence of DKK3, resulting in phosphorylation of DSH, which prevents GSK3 from degrading -catenin.

The link between both the WNT & ER signalling pathways, particularly via Polycomb protein EZH231, it has been suggested that DKK’s involvement of WNT signalling activity can relate forward into the ER dependent pathway (and vice versa) to strengthen survival and growth with DKK3 promoter methylation being associated with positive ER status.5-azacytidine and trichostatin A, for example, have been shown to restore DKK3 expression *in vitro* (Figure 2). In the clinic, however, attempts to re-establish ER expression with hypomethylating medications have failed, EMT influences the polarisation of mammary cells, milk flow patterns, particularly during pregnancy and during wound healing, cell movements are important. Mediated by ZEB1, SNAIL, and TWIST, among other transcription factors (TFs). SNAIL, for example, activates the Methyltransferase DNMT1 and inhibits CDH1 through DNA methylation. Furthermore, TGF-induced EMT modulates SNAIL transcription reactivation via the H3K27me3 demethylase KDM6B. Increased levels of SNAIL and KDM6B have been associated to cancer recurrence, metastases, and poor flatline survival in invasive breast carcinomas (Beatson, 1896; Liu et al., 2006; Saez-Ayala et al., 2013; Hanker et al., 2020). As a result, one can expect that targeting H3K27me3 demethylases in combination with DNA hypomethylating medicines, which are prospective treatment targets in other solid tumours such as castration-resistant prostate cancer, could reduce recurrence (Liu et al., 2001).

### Polycomb complexes and HH signalling

Epigenetic changes in breast cancer impair HH signalling, which is an important developmental pathway. Cancer progression is driven by increased ligand-dependent pathway activation and unregulated cell division (Early Breast Cancer Trialists’ Collaborative Group, 2005). Whenever the promoters of the SHH, HH Ligand, or its subsequent receptors, PTCH, is hypomethylated, the pathway is activated more ligand-dependently, resulting in uncontrolled cell division and cancer progression (Early Breast Cancer Trialists’ Collaborative Group, 2005). HH signalling also helps to promote normal and tumorigenic breast stem cells by boosting the production of PCGF4 (BMI1), a constituent of the PRC1 complex (Jeselsohn et al., 2015). Breast cancer stem cells have already been related to hormonal therapy resistance; however, it is unclear whether the appearance of stem cell like features in resistant cells is attributable to the multiplication of pre-existing highly specialised tumour cells or to epigenetic modifications that promote dynamic reprogramming (Jeselsohn et al., 2015).

According to the findings, combining medicines that directly block HH signalling with epigenetic modifiers like DNMTs to recover HH antagonistic control could alter cancer stem cell viability and differentiation. It has previously been reported on the utilisation of two-step approaches that combine several classes of medicines to trigger a process known as targeted phenotypic flipping to treat resilient melanoma cells to lineage-specific therapy (Razavi et al., 2018). WNT and Hedgehog signalling pathways are required for the development of embryonic mammary glands, and their activation must be carefully coordinated spatially and temporally (SHH). In healthy mammary epithelial cells, DKK3 binds to LRP, a Frizzled WNT pathway coactivator, preventing the route from becoming activated in the vicinity of the WNT ligand. E-Cadherin attaches to cytoplasmic -catenin in the lack of WNT activation, which is eliminated by GSK3.

The oncogenic E2-ER axis is the focus of endocrine treatment. The very first-time steroid hormonal signalling being linked to breast tumor progression when both ovaries were surgically removed from patients with breast cancer, resulting in tumour regression and pave the way for endocrine therapy (Hanker et al., 2020). Treatments that decrease estrogen synthesis as well as techniques that specifically target the estrogen receptor are used in hormonal therapy, which is really the benchmark for ER + breast cancer (ER). Selective oestrogen receptor degraders (SERDs), Selected Oestrogen Receptor Modulators (SERMs), and Aromatase Inhibitors are the three categories (AIs) (Fanning et al., 2016). In addition, next-generation ER targeting medications are presently being investigated in ER+/HER2 metastatic melanoma as adjuvant therapy or in combination with other therapy (Helleman et al., 2008).

### Mechanisms Of endocrine therapy resistance, as well as possible alternatives

Considering the fact that the endocrine therapy is effective in the treatment of ER + breast cancer patients, resistance develops in around 25% of early-stage patients and virtually all metastatic patients, results in a poor clinical prognosis (Liu et al., 2006; Serrano-Gomez et al., 2016; Bell et al., 2019); (Jansen et al., 2005; Generali et al., 2006; Pontiggia et al., 2009). Resistance to endocrine therapy has been classified as either intrinsic or acquired. Patients with breast cancer frequently experience clonally distinct progression as a result of the selection of genetic changes under treatment (Folgiero et al., 2008).

### Resistance mechanisms associated with the tumor microenvironment and the host

The tumour microenvironment’s role as a regulator of these pathways and contribution to endocrine responsiveness has recently been established. This theory has been supported by studies including gene expression studies and biomarkers linked to hormonal therapy outcomes (Encarnacion et al., 1993; Brinkman et al., 2010) as well as the more complex *in vitro* or *in vivo* existing experimental systems (Gutierrez et al., 2005). Endocrine resistance is linked to stromal cells (endothelial, fibroblasts and immune cells), structural features of the microenvironment and soluble substances (e.g., interleukins and growth factors) as well as other micro-environmental variables including hypoxia and acidity (Lopez-Tarruella and Schiff, 2007).

The role of tumorigenic cell pathways in modulating these microenvironmental and extracellular stimulus has previously been characterised (Munzone et al., 2006; Xia et al., 2006), inferring potential signalling components (e.g., SRC Kinase/integrin/FAK) that might be targeted to overcome endocrine resistance (Osborne et al., 2003; Shi et al., 2009). In addition, the list of other host genome–associated variables influencing endocrine sensitivity is rising as a consequence of new pharmacogenomic and high-throughput research.

### Resistance mechanisms associated with tumors

However, as previously indicated, the most of pathways that may play a role in endocrine resistance originates in tumour cells. These pathways can be split into three categories, each of which has components and mechanisms that overlap.

The two different types of ER regulator are ER and ER coregulators. The first group includes the ER, with coregulators, as well as other factors that alter the normal ER activity and modify receptor functions in relation to endocrine therapy. In refractory endocrine cancers, reduction of ER synthesis (i.e., the ER isoform) culminates in an endocrine-insensitive phenotype, that is rare (Lavinsky et al., 1998; Shou et al., 2004; Musgrove and Sutherland, 2009). Treatments that block growth factor receptors pathways that are known for down regulation, ER can enhance ER expression and endocrine sensitivities both in experimental and clinical situations (Schiff et al., 2004; Levin and Pietras, 2008; Santen et al., 2009) Reduced endocrine responsiveness has also been associated to the expression of various ER splicing variation, specifically the recently found minor variance ER36 (Musgrove and Sutherland, 2009) and oestrogen-related receptors. Furthermore, statistics suggest that ER coregulators, whether negative (corepressors) or positive (coactivators), have a role in defining endocrine sensitivity and resistance by influencing the balance of agonistic vs. antagonistic SERM activity. Both in clinical and experimental contexts, dysregulation of a ER co-activator AIB1 (also abbreviated as SRC3 or NCoA3) has been associated to tamoxifen resilience (Span et al., 2003; Butt et al., 2005), while reduced expression of the co-repressor NCoR has been detected in tamoxifen-resistance experimental malignancies (Arpino et al., 2008). The ER as well as its coregulators are heavily influenced by posttranslational modifications. Growth factor receptor [e.g., FGFR (fibro-blast growth factor receptor, EGFR/HER2, and IGF1-R ) and other cellular and stress-related kinases [e.g.p42/44, JNK , AKT, and PKA (protein kinase A and p38 MAPKs ), PAK1] regulate several posttranslational modifications (p21-activated kinase). Ubiquitination, Methylation, Phosphorylation, and other posttranslational modifications of ER and its co-regulators have been identified to alter ER activity and susceptibility to various endocrine therapies (Chakraborty et al., 2010) Outside of the nucleus, ER interfaces with cytoplasmic and membrane signalling complexes to activate and regulate a variety of growth factor receptors as well as other cell - signalling cascades (Kern et al., 1994; Morgan et al., 2009; Shi et al., 2009; Spoerke et al., 2016).

### Cell cycle signalling molecules

Molecules associated in cellular and biological responsiveness to endocrine therapy, such as cell growth inhibition and apoptosis induction, are included in endocrine resistance–related pathways. The majority of information on the participation of these pathways comes from preclinical investigations. Positive cell-cycle regulators, notably those directing G1 phase progression, and also negative cell cycle regulator, have both been demonstrated to disrupt and decrease endocrine therapy’s antiproliferative action, tends to result in resistance (Fribbens et al., 2016). Endocrine resistance is caused by overexpression of positive cell-cycle regulators MYC & cyclins E1 and D1, which activate cyclin-dependent kinases (CDKs) for G1 phase or reduce the inhibitory effects of negative cell-cycle regulators (p21 & p27) (Gates et al., 2018).

### Growth factor receptor pathways

In the case that the ER system is effectively inhibited, the third set of regulatory mechanisms in endocrine resistance would comprise those that can provide alternate proliferation and migration inputs to tumours. Importantly, through bi-directional interactions and control of the ER, these mechanisms—such as growth factors and other cellular-kinase pathways—might be able to offset the inhibitory activity of endocrine therapy. Many of these pathways, on the otherhand, it might develop into ER-independent drivers of tumour development and survival, making patients susceptible to all kinds of endocrine therapy, either early or later on. It has been suggested that fibroblast growth factor (FGF), insulin/IGF1 receptors HER, tyrosine kinase receptors, and vascular endothelial growth-factor (VEGF) receptors are all involved (Harrod et al., 2017; Jeselsohn et al., 2018; Morel et al., 2020).

Alteration Of ESR1 And Genes Involved In Estrogen-Mediated Signalling.

The tumour cell’s reliance on ER for growth and survival is targeted by endocrine treatment. As a result, bypassing pharmacological inhibition relies on the accumulation of changes in the ER and its downstream targets. The main mechanism of resistance in most cases is ligand independent ER reactivation (Oronsky et al., 2014). Constitutive ER activation can be mediated by mutations in the ESR1 gene (which codes for ER) and is a major driver of acquired resistance. The majority of ER mutations occur in the LBD at two neighbouring amino acids: tyrosine at position 537 transformed to asparagine, cysteine, or serine (ERY537 N/C/S) and aspartic acid at position 538 altered to glycine (ERD538G). From a structural standpoint, these changes remain stable ER in an agonists configuration, resulting in constitutively active state (Oronsky et al., 2014). ESR1 mutations are detected in less than 1% of original tumours, however they are reported in 20–40% of tumours after endocrine therapy and have been associated to poor AI & tamoxifen efficacy (O’Neil et al., 2017; Zucchetti et al., 2019; Xu et al., 2020). The nearly complete detection of ESR1 alterations in hematologic malignancies after the AI therapy shows that under the constraints of endocrine treatment, uncommon, resistant clones can be selected.

Several studies have focused on establishing new therapeutic strategies for tumor tissues with ESR1 mutations in recent years. Continuous ER signalling encourages hormone independent development and thus is linked to a distinct transcription network involved in signaling pathways and metastasis as a result of this process (Fukumoto et al., 2018). Activating kinases, epigenetic modifying enzymes and ER co-regulators, are required for the development of ESR1 mutants (Fukumoto et al., 2018). .As a consequence, they could be employed to treat ESR1 mutant malignancies in the preclinical stage. Another type of genetic mutation discovered in metastatic ER + breast cancer is ESR1 gene fusion events, which are likely to represent novel resistance drivers. As a result of ESR1 chromosomal rearrangement occurrences, the ER’s LBD is replaced by another protein.

### Endocrine-resistant breast cancer is caused by epigenetic factors

According to a whole-genome sequencing study, epigenetic factors are among the most commonly changed factors in human malignancies. The most frequent genetic modifications in many types of cancer are inactivating mutations as well as the loss of SNF/SWI subunits. In breast cancer, ARID1A impacts breast luminal lineage adherence and sensitivity to endocrine treatment.

Patients’ poor response to SERDs suggests that ARID1A loss-of-function mutations are more frequent in endocrine-resistant metastatic situations, implying that they can also cause endocrine resistance.

ARID1A deficiency affects chromatin accessibility and transcription factor binding, as well as the binding of ER and FOXA1 to chromatin, all of which influence luminal cell destiny.

According to Xu et al., long-term ER suppression could result in the generation of individuals with ARID1A inactivating mutation, promoting a luminal-to-basal phenotypic transition (Bitler et al., 2015). In the clinic, ER + tumours cured with endocrine therapy, reduce ER expression, by becoming resistant to hormone therapy. The increasing prevalence of ARID1A mutation in endocrine resistant breast cancer, including its prevalence in other cancers, highlights the need of treating ARID1A mutant tumours with targeted therapeutic strategies (Morel et al., 2020).

One of the therapy paradigms examined in ARID1A mutant cancers is synthesised lethality, which relates to the lethal consequence of simultaneous alteration of two genes which, when separately disrupted, do not impact cell viability (Morel et al., 2020). For example EZH2 suppression and ARID1A mutations are synthetically fatal in ovarian cancer, and HDAC2 inhibition amplifies this effect. In ARID1A defective cells, HDAC2 is attracted to EZH2/ARID1A co-target genes including such PIK3IP1, a PI3K/AKT signalling inhibitor, leading in incorrect stimulation of this mitogenic system (Shiino et al., 2016). These two processes, ARID1A loss of function and enhanced PI3K/AKT signalling, are typically detected in endocrine-resistant breast cancer cells (Shiino et al., 2016). As a consequence, one can believe that targets EZH2 in breast cancer patients with ARID1A mutations might be a promising treatment option.

### Epigenetic avenues in the endocrine therapy

In the realm of endocrine therapy, there are a variety of epigenetic options. Despite the fact that endocrine and molecularly targeted have been shown to cure the great majority of breast cancers, they have failed to target a tiny percentage of the population, leading to recurrence and therapeutic resistance. Compounding variables like tumour genetic instability enables tumours to adapt to a range of stresses, including the selective pressure produced by therapeutic drugs, which we addressed previously. As a result, precise patient classification and personalised treatment approaches would be required to reduce the significant morbidity and mortality of individuals with ER + metastatic breast cancer (Shiino et al., 2016).

Tumorigenesis and medication resistance are both influenced by epigenetic instability. Epidrugs have primarily been used to treat haematological malignancies, with limited efficacy in solid tumors. The failure to treat solid tumours, on the other side, can be traced back to a one size fits all approach. Epigenetic reprogramming’s plasticity enhances cancerous cells’ overall fitness, making individualised cancer treatment much more challenging. According to multiple preclinical and clinical studies, epidrugs have synergistic benefits with a number of therapeutic methods, including immunotherapy, radiation, and endocrine therapy. One of the most notable areas of current drug discovery operations is the development of small compounds that target chromatin regulators (Shiino et al., 2016).

The majority of the studies focused on epigenetic changes that occur when cancer progresses and resistance develops. Small molecule HDACi blockers (vorinostat and entinostat ) and also DNA hypomethylating drugs (decitabine & 5-azacytidine) have been explored as re-sensitizing strategies to endocrine therapy in ER + preclinical models. The modes of action of DNMT inhibitors (DNMTs) have been proposed as de-methylation of tumour suppressor genes and an unique viral mimicking mechanism. Furthermore, in endocrine-resistant breast cancer, epigenetic dysregulation is a prevalent occurrence. For example, in roughly 20% of patients that continue through tamoxifen treatment, promoter hyper-methylation of ESR1 causes loss in ER expression (Shiino et al., 2016). In ER human breast cancer cells, letrozole (AI) and entinostat (HDACi) and can re-establish ER & aromatase expression, resulting in growth suppression.

## Conclusion

New findings, as with all parts of science, produce new questions, and some of the most important unanswered questions like. Is it possible to use the dynamic nature of epigenetic modifications to develop short-term treatment techniques to avoid selection toward a resistant phenotype, or are epidrugs’ underlying processes contributing to resistance formation is possible to follow disease progression and therapy response using epigenetic markers. Endocrine therapy has been proven to be an important treatment option for hormone-responsive breast cancers. However, there is still an urgent need to develop strategies to combat the phenotype of resistance that appears to be unavoidable. Recent epidrug breakthroughs attest to the developing new era of epigenetic-based treatments for screening and treating a variety of disorders, including breast cancer.
